# Glucocorticoid-induced diabetes mellitus: mechanisms, risk factors, and clinical pathways with insights from autoimmune rheumatic diseases

**DOI:** 10.1007/s00296-026-06099-z

**Published:** 2026-03-26

**Authors:** Maria Soura, Amalia Bakiri, George E. Fragoulis, Konstantinos D. Vassilakis

**Affiliations:** 1 Joint Academic Rheumatology Program, First Department of Propaedeutic and Internal Medicine, School of Medicine, National and Kapodistrian University of Athens, Athens, Greece; 2 School of Infection and Immunity, University of Glasgow, Glasgow, UK

**Keywords:** Diabetes mellitus, Glucocorticoids, Hyperglycemia, Rheumatic diseases, Autoimmune diseases

## Abstract

Diabetes mellitus (DM) is characterized by persistent hyperglycemia due to impaired insulin secretion, action, or both. Glucocorticoid-induced DM (GIDM) is a clinically significant subtype, especially in rheumatology, where glucocorticoids (GCs) are widely used for the management of autoimmune rheumatic diseases (ARDs). GCs increase gluconeogenesis, reduce insulin sensitivity, impair β-cell function, and alter adipokines and hypothalamic signaling, promoting hyperglycemia. Oral GCs carry the greatest risk, though intra-articular and intramuscular injections can also cause dysglycemia. Risk factors include older age, higher body mass index (BMI) and central adiposity, hypertriglyceridemia, family history of DM, higher GC dose and treatment duration, and disease activity. In non-ARD populations, GIDM occurs in 15–52% of GC-treated individuals, while in ARDs, rates vary by disease. In Systemic lupus erythematosus, occurrence ranges from 10–26% and, beyond classic metabolic risk factors, higher disease activity and damage scores, higher GC doses, and mycophenolate mofetil are associated with increased risk, whereas hydroxychloroquine seems to be protective. In Rheumatoid arthritis, true incidence remains uncertain, though GC dose, especially > 10 mg/day, and prolonged duration correlate with increased risk. In Polymyalgia rheumatica and Giant cell arteritis, GIDM increases dose-dependently, with an occurrence of 6% and 13%, respectively. Evidence for other ARDs is limited. Management of GIDM should be guided by a multidisciplinary approach, aiming for a personalized therapeutic strategy. This review summarizes current knowledge on the mechanisms, epidemiology, and risk factors of GIDM in ARDs, aiming to raise awareness within the rheumatology community, highlight key gaps in the literature, and outline implications for future research.

**Supplementary Information:**

The online version contains supplementary material available at 10.1007/s00296-026-06099-z.

## Introduction

Diabetes mellitus (DM) is a chronic metabolic disorder characterized by hyperglycemia resulting from defects in insulin secretion, insulin action, or both. While type 1 (T1DM) and type 2 (T2DM) DM dominate the clinical landscape, other less prevalent subtypes, such as glucocorticoid-induced diabetes mellitus (GIDM), remain relatively under-investigated [1]. Glucocorticoids (GCs) are widely used as first-line treatment or adjuvant therapy for various conditions due to their anti-inflammatory and immunosuppressive properties, despite their adverse effects [2, 3]. In rheumatology, where prolonged or high-dose GC therapy is often used for disease control, recognizing and understanding GIDM is critical for optimizing outcomes. This narrative review provides a critical synthesis of current knowledge on the pathophysiological mechanisms, prevalence, and risk factors of GIDM in patients with autoimmune rheumatic diseases (ARDs).

## Methods

A literature search for this narrative review was conducted using PubMed to identify relevant studies published in English from inception up to June 2025. The following keywords/terms were used: “steroid-induced diabetes mellitus” OR “steroid-induced hyperglycemia” OR “glucocorticoid-induced diabetes mellitus” OR “glucocorticoid-induced hyperglycaemia” OR “corticosteroid therapy” OR “glucocorticoid therapy” OR “SIDM” OR “GIDM” AND “autoimmune rheumatic diseases” OR “ARDs” OR “immune-mediated inflammatory diseases” OR “rheumatic diseases” OR “inflammatory rheumatic diseases” OR “rheumatoid arthritis” OR “RA” OR “systemic lupus erythematosus” OR “SLE” OR “antiphospholipid syndrome” OR “APS” OR “vasculitis” OR “large-vessel vasculitis” OR “giant-cell arteritis” OR “GCA” OR “ANCA-associated vasculitis” OR “spondyloarthritis” OR “SpA” OR “psoriatic arthritis” OR “PsA” OR “axial spondyloarthritis” OR “AxSpA” OR “polymyalgia rheumatica” OR “PMR” OR “connective tissue diseases” OR “systemic sclerosis” OR “SSc” OR “Sjögren’s syndrome” OR “pSS” OR “idiopathic inflammatory myopathies” OR “myositis” OR “dermatomyositis”.

Studies were initially screened based on their titles and abstracts to determine relevance, followed by full-text review. Additionally, the reference lists of the selected articles were examined to identify further studies that may have been missed in the primary search.

To be eligible for this review, studies had to investigate GCs use in relation to glucose metabolism or DM presentation, specifically within individuals having ARDs. Original research articles, randomized controlled trials, or meta-analyses published up to June 2025 were included. Studies were excluded if the full text was not accessible or focused on populations having diseases other than ARDs, or on non-steroid-related causes of DM.

### Glucocorticoid-induced diabetes mellitus

GC use is associated with significant metabolic complications, including impaired glucose tolerance and decreased insulin sensitivity often leading to glucocorticoid-induced hyperglycemia (GIH) and GIDM [4]. GIH refers to worsening glycemic control in the context of pre-existing DM associated with exogenous GC therapy, whereas GIDM describes new-onset DM as a direct consequence of GC exposure in individuals without a previous diagnosis of DM [5].

GIDM is diagnosed using standard DM criteria, including fasting plasma glucose ≥ 126 mg/dL (≥ 7 mmol/L), random plasma glucose ≥ 200 mg/dL (≥ 11.1 mmol/L), or a 2-h plasma glucose ≥ 200 mg/dL following an oral glucose tolerance test (OGTT), provided these irregularities occur after the initiation of GC therapy [6]. However, testing should account for the diurnal glycemic pattern induced by GC use (especially intermediate-acting GC), which disproportionately increases postprandial and evening glucose [7]. As a result, relying completely on morning fasting glucose measurement as a diagnostic criterion may underestimate the true incidence of GIH or GIDM [7]. Similarly, OGTT may not be optimal for GIDM diagnosis due to its fasting requirement and potential underestimation of glucose elevation occurring primarily in the evening. Yet, in high-risk, non-diabetic individuals, blood glucose measurement and OGTT should be performed early on during the monitoring of the patients to help detect underlying DM susceptibility [8].

GIDM most commonly emerges between the 2nd and 4th week of GC therapy, supporting continuous monitoring, especially for individuals experiencing hyperglycemia or requiring antidiabetic medications [9]. Individuals with pre-existing DM or risk factors for GIDM should be screened even when low GC doses are prescribed [10]. Typically, GIDM improves following dose tapering or GC discontinuation, but in some cases, it may persist [6].

### Pathophysiology

Multiple tissue-specific effects of GCs have been described contributing to the development of GIDM, with most of these pathways mediated by the GC-receptor, binding to specific target genes and altering their expression [7]. Thus, changes in liver, skeletal muscle and adipose tissue primarily mediate GIDM and GIH pathophysiology, whereas other organs such as the pancreas, the hypothalamus and the bones further disrupt the glucose regulation [7].

#### Liver

Following GC therapy, elevated hepatic glucose production, insulin resistance and hepatic steatosis have been described [7]. GCs induce hepatic gluconeogenesis by upregulating enzymes of de novo glucose synthesis, which is further amplified by the uptake of gluconeogenic precursors from the GC-mediated protein catabolism in skeletal muscles and lipolysis in adipose tissue [7, 11, 12]. Hepatic insulin resistance is also developed, as GCs impair the phosphorylation of downstream second messengers of the insulin cascade, leading to unopposed gluconeogenesis [13–15]. Despite that, the pro-lipogenic effect of insulin is preserved [16], which in the setting of compensatory hyperinsulinemia (due to GIH), increases de novo hepatic lipogenesis leading to steatosis and exacerbation of hepatic insulin resistance [17, 18]. Other factors, such as the elevated liver uptake of circulating non-esterified fatty acids (NEFAs) and the increased ceramide synthesis, are also associated with hepatic dysregulation leading to GIDM [17–19].

#### Skeletal muscle

GCs decrease skeletal muscle insulin sensitivity and glucose uptake, by inhibiting the insulin-dependent upregulation of glucose transporter 4 (GLUT4) to the cell surface of skeletal muscles [20]. Moreover, long-term GC therapy leads to myopathy [21], by promoting proteolysis and diminishing protein synthesis [11]. The elevated amino acids indirectly impede insulin-induced glucose transport and glycogen synthesis in muscle tissue [22], whereas the also act as substrate for hepatic gluconeogenesis [7].

#### Adipose tissue

Adipose tissue actively participates in GIDM pathogenesis, via decreased glucose uptake, increased triglyceride uptake and synthesis, and increased lipolysis providing substrates for hepatic gluconeogenesis [7]. Similarly to endogenous hypercortisolemia [23], exogenous GCs lead to lipid redistribution from peripheral to abdominal adipose depots [24]. This depot-dependent adipose tissue breakdown and expansion results in central obesity, which is linked to insulin resistance and T2DM, due to perihepatic white adipose tissue (WAT) hypertrophy and direct drainage of NEFAs and pro-inflammatory factors into the portal circulation [7, 9, 25–27]. Moreover, despite promoting adipose expansion (via insulin dependent lipogenesis, triglyceride synthesis and WAT hypertrophy [28, 29]), GCs concurrently increase lipolysis by upregulating the hormone-sensitive lipase and monoacylglycerol lipase [29, 30]. This increases the serum levels of NEFAs and glycerol [31, 32], leading to hyperglycemia due to hepatic gluconeogenesis and impairment of liver and muscle insulin sensitivity from ectopic lipid accumulation [7]. Furthermore, GCs indirectly affect both adipose tissue and whole-body insulin sensitivity through the regulation of adipokine levels secreted by WAT [4, 7, 28].

#### Pancreas, Bone tissue and central nervous system

Pancreatic β-cell dysfunction has been previously linked to GIDM [7]. In susceptible individuals, short-term GC administration is associated with decreased β-cell insulin secretion and whole-body glucose disposal [33–35]. Normally, glucose uptake by β-cells causes an increase in intracellular ATP levels, leading to closure of the ATP-sensitive potassium channels and eventually depolarization of the cell membrane. Thus, voltage-gated channels are activated resulting in rapid calcium influx and eventually insulin release from the β-cells [7, 36]. GCs reduce insulin secretion by disrupting this process in multiple stages, such as glucose uptake, membrane de- and re-polarization and α2-adrenergic signaling [37–39]. Additionally, other mechanisms of pancreatic β-cell dysfunction have been described, such as direct inhibition of insulin synthesis and GC-induced cytotoxicity via reactive oxygen species leading to β-cell apoptosis [7, 40, 41].

When it comes to bone tissue, GC-osteoporosis is associated indirectly with GIDM development [7]. Osteoporosis following GC therapy is caused by suppression of the bone forming osteoblasts and activation of the bone-resorbing osteoclasts [42]. In addition, GCs also contribute to GIDM by increasing leptin secretion from adipose tissue, which in turn suppresses the expression of osteocalcin, thereby indirectly inhibiting insulin secretion [7, 43].

**Fig. 1:**
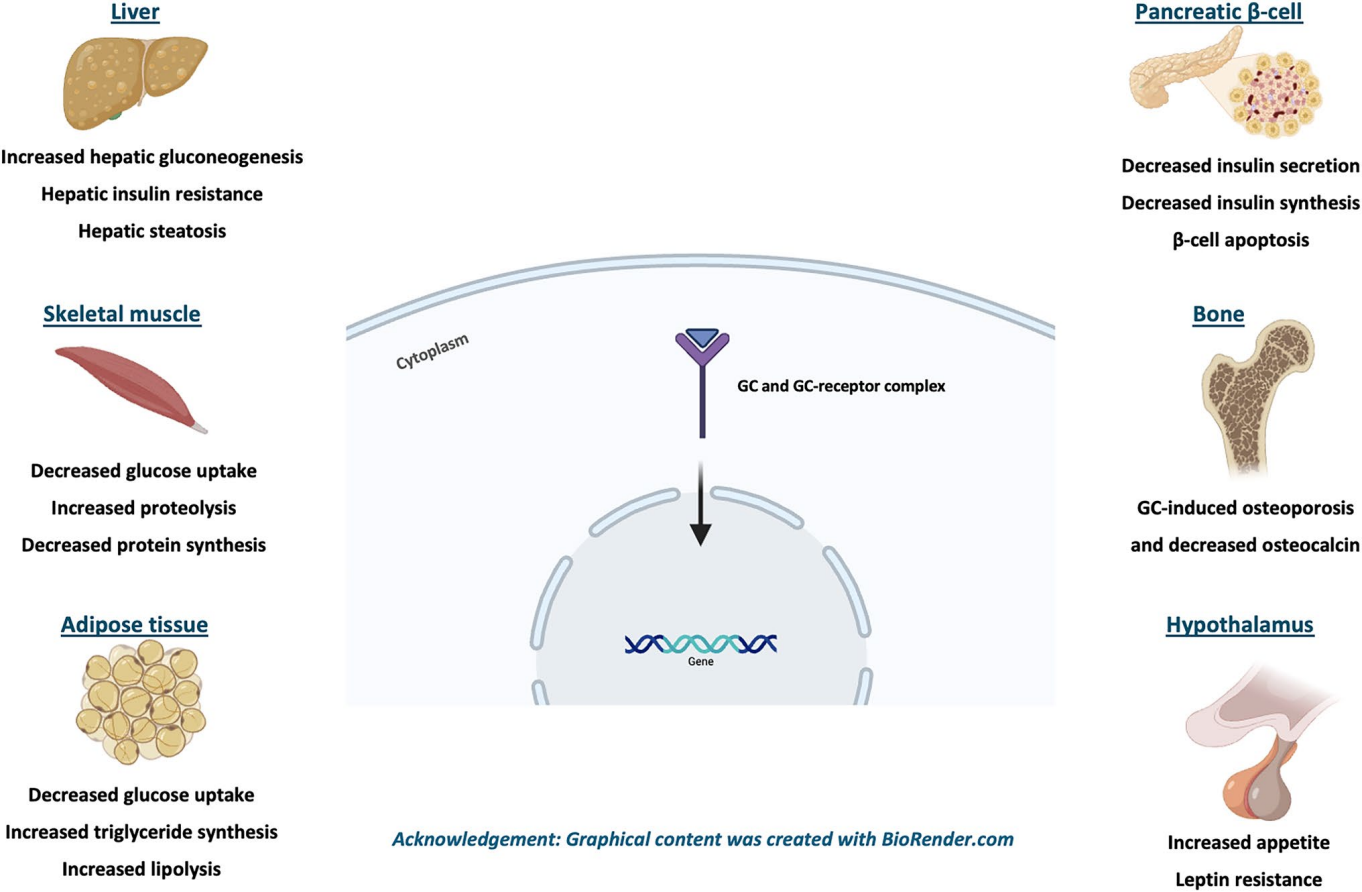
Multi-organ pathophysiological aspects of GIDM. Schematic overview of GC effects across liver, skeletal muscle, adipose tissue, pancreatic β-cells, bone, and hypothalamus. The GC-receptor complex mediates transcriptional messages that: (i) increase hepatic gluconeogenesis and de novo lipogenesis, leading to hepatic insulin resistance and steatosis; (ii) reduce GLUT4-dependent glucose uptake and enhance proteolysis, while decreasing protein synthesis in skeletal muscle; (iii) promote visceral adiposity with concurrent lipogenesis and lipolysis in adipose tissue that aggravate systemic insulin resistance; (iv) decrease β-cell insulin synthesis and secretion, and promote β-cell apoptosis; (v) contribute to osteoporosis and reduced osteocalcin signaling; and (vi) increase leptin resistance and appetite. Collectively, these pathways shift glucose homeostasis toward hyperglycemia and GIDM. *GC* Glucocorticoids, *GIDM* Glucocorticoid-induced diabetes mellitus, *GLUT4* Glucose transporter type 4

Finally, the effects of GCs on the central nervous system (CNS) should be also considered in GIDM pathogenesis. Under physiologic conditions, appetite is mediated by orexigenic peptides, such as neuropeptide Y (NPY) and agouti-related peptide (AgRP), which are released by neurons in the arcuate nucleus of the hypothalamus [7]. Leptin is one of the hormones responsible for the appetite regulation by suppressing NPY–AgRP neurons and thus, the release of orexigenic peptides [44]. GCs upregulate the expression and release of these peptides, whereas they also synergistically antagonize the action of leptin in the hypothalamus by directly reducing leptin-dependent JAK–STAT signaling [7, 45–47]. Despite that, GCs shift nutritional preference towards high-fat and/or high-sugar foods, known as “comfort food”. Therefore, GCs promote a hypercaloric diet, which indirectly results in obesity and DM [7]. The pathophysiological aspects of GIDM are further depicted in Fig. 1.

### GCs nomenclature and route of administration

Accurately estimating the risk of GIH or GIDM when administering GCs remains challenging due to the heterogeneity of dosing regimens, pharmacologic potency, routes of administration and treatment duration used across different clinical conditions [7].

GCs are commonly classified according to their duration of action and relative potency [48]. Short-acting agents, like hydrocortisone and cortisone, have a half-life of less than 12 h [7, 10, 48, 49]. Intermediate-acting GCs, such as prednisone, prednisolone and methylprednisolone demonstrate half-lives of 16–36 h, whereas long-acting agents, including betamethasone and dexamethasone, have half-lives ranging from 36 to 54 h [7, 10, 48, 49]. Among these, the intermediate-acting GCs are the most frequently used, being 4–5 times more efficacious than the short-acting, while long-acting agents may reach up to 25-fold greater potency [7, 10, 48]. In terms of dosage, low-dose GCs are defined as prednisolone equivalent dose (PED) less than 7.5 mg/day, medium-dose regimens range from 7.5 mg to 30 mg/day, and high-dose from 30 to 100 mg PED/day [50, 51]. When the GCs exceed 100 mg PED/day they are considered very high dose [51]. For pulse therapy, the therapeutic regimen consists of at least 250 mg PED/day for one or a few days [51].

GCs can be administered via multiple routes. Although all common routes of GC administration, have been associated with a higher GIH risk, when given in high doses [7, 52–55], oral GCs pose the greatest risk, regardless of the dosing regimen [7, 56]. Intra-articular (IA) and intramuscular (IM) injections, which are commonly used in ARDs, have also been correlated with GIH development [57, 58], but factors such as differences in GC injection release profile and previously established good glycemic control appear to influence glucose homeostasis following these regimens [58–60]. In a post-hoc analysis of a phase 2 study enrolling 33 patients with knee osteoarthritis and T2DM, individuals receiving extended-release triamcinolone acetonide IA injections (n = 18) versus immediate-release triamcinolone acetonide (n = 15) were compared [61]. The former had lower median change from baseline in maximum glucose level, less median time with a glucose level > 250 mg/dL, a smaller proportion of patients with a maximum glucose level of > 250 mg/dL, and a greater percentage of time in the target glucose range [61]. In another study, evaluating the effects of IA injection of betamethasone acetate/betamethasone sodium phosphate on blood glucose levels in controlled DM individuals (glycated hemoglobin HbA1c < 7) with symptomatic knee osteoarthritis, no significant effect on serum fructosamine levels were observed (measured just prior and 2 weeks following the injection), whereas only transient increases in blood glucose levels were reported [58].

### Epidemiology of GIDM in non-ARDs

GIDM is a clinically important complication of GC therapy affecting approximately 15–52% of individuals treated with GCs [9, 26, 27, 62–67]. The occurrence of GIDM depends on various factors, such as advanced age, higher BMI, abdominal obesity, hypertriglyceridemia and family history [7, 67], as well as the disease itself. In a retrospective study of individuals with respiratory diseases treated with prednisolone (GC equivalent > 20 mg/day), 15% developed GIDM [64]. Likewise, a study, enrolling 90 patients on GCs as a part of their cancer treatment, reported GIDM in 19% [62], while similar findings have been described in pancreas transplant recipients [65]. Among patients with pemphigus vulgaris treated with GCs, the incidence of GIDM has been reported at 22% in two different studies [26, 27]. A notably higher rate has been observed among GC treated individuals with primary renal diseases, reaching approximately 40% [63]. Along the same lines, similar incidence has been reported in a prospective study enrolling noncritically ill individuals recently diagnosed with hematologic malignancies initiating high-dose GC therapy [9]. A slightly higher occurrence (41%) has been described in individuals with secondary adrenal insufficiency receiving hydrocortisone or cortisone acetate [67]. The highest incidence has been recorded in a cohort of individuals with neurologic conditions, including neuroinflammatory diseases, neuropathies and polymyositis, receiving prednisolone (average dose of 42 mg/day) with 50% of the cases developing GIDM [66].

### Glucocorticoids in ARDs

Despite the availability of new treatments, GCs continue to be central to the management of ARDs. In Rheumatoid arthritis (RA), short-term GCs are used when initiating or changing conventional synthetic disease modifying antirheumatic drugs (csDMARDs), in different dose regimens and routes of administration, but recent recommendations underline the significance of tapering and discontinuation, as rapidly as clinically feasible [68]. In Systemic lupus erythematosus (SLE), GC therapy is based on the type and severity of organ involvement [69]. Intravenous methylprednisolone pulses (125–1000 mg/day, for 1–3 days) are frequently used in life-threatening organ involvement, followed by oral tapering regimens aiming for a maintenance dose of ≤ 5 mg/day PED and when possible, complete withdrawal [69]. High-dose GC therapy (40–60 mg/day PED) is initiated immediately for induction of remission in active Giant cell arteritis (GCA) or Takayasu arteritis (TAK) [70], as well as in ANCA-associated vasculitis [71]. For individuals with Polymyalgia rheumatica (PMR) the minimum effective individualized GC dose and duration is typically used (within a range of 12.5–25 mg PED), followed by slow tapering over months to avoid relapse [72]. In Sjögren’s syndrome (pSS), GCs are used at the minimum dose and length of time necessary [73]. In Spondyloarthritis (SpA), systemic GC therapy is not usually administrated, rather than in some selected cases (e.g., persistent polyarticular forms and/or as bridging therapy), whereas injections directed to the local site of musculoskeletal inflammation can be used as adjuvant therapy [74, 75].

### GIDM in ARDs

#### Systemic lupus erythematosus

SLE is a chronic ARD characterized by significant heterogeneity [76] and it can affect multiple organ-systems. Pharmacological treatment of SLE ranges from hydroxychloroquine (HCQ) to highly potent immunosuppressive medications depending on disease activity and organ involvement [69]. GCs are central in SLE treatment due to their effectiveness in controlling disease exacerbations [77].

A relatively understudied complication is GIDM with a recent study indicating a higher, dose-dependent risk in SLE individuals treated with GCs compared to non-users [Current use vs Non-use; adjusted Hazard Ratio (aHR): 2.71, 95% CI: 2.05–3.57/ daily dose 5.0–14.9 mg—aHR: 2.67, 95% CI: 1.94–3.68/ daily dose ≥ 25 mg- aHR: 6.63, 95% CI: 3.55–12.38] [55]. Regarding GIDM occurrence, rates vary from 10 to 26% (Table 1) [78–81]. A case–control study evaluated the occurrence of GIDM in 358 individuals with no previous history of DM receiving high-dose GC induction therapy for lupus nephritis [78]. Within the first year of follow-up, nearly 10% of the patients met the criteria for GIDM, while another 25% developed prediabetes [78]. In a different study evaluating 127 SLE patients receiving high-dose prednisolone (≥ 1 mg/kg/day), the incidence of GIDM was found at 13% (95% CI: 6.8–18.4) [79]. Finally, a much higher occurrence of GIDM was observed in a cross-sectional study of 81 SLE patients, without prior DM diagnosis, treated with prednisolone (≥ 1 mg/kg/day), reaching 26%. After 3 years of follow up, 11% of the cases continued to meet the diagnostic criteria for DM [81].

**Table 1:** Occurrence and risk factors for GIDM across ARD populations

Population	GIDM Occurrence	Risk Factors
General Population	15–52%	1. Advanced age 2. Higher BMI 3. Abdominal obesity 4. Hypertriglyceridemia 5. Type of disease
SLE	10–26%	1. Classic metabolic risk factors* 2. Disease-specific factors** 3. Prednisone dose ≥ 1 mg/kg/day 4. Concurrent use of MMF
RA	7–24%	1. Daily GC dose^◊^ 2. Duration of GC therapy 3. Classic metabolic risk factors
PMR & GCA	PMR: 6%, GCA: 13%	Daily GC dose^∇^
Vasculitis	Increased risk^∇∇^	Daily GC dose^∇^

*ARD*: Autoimmune rheumatic diseases; *aHR*: adjusted hazard ratio; *BMI*: Body mass index; *GC*: Glucocorticoids; *GCA*: Giant-cell arteritis; *HR*: Hazard ratio; *METS-IR*: Metabolic score for insulin resistance; *MMF*: Mycophenolate mofetil; *PED*: Prednisolone equivalent dose; *PMR*: Polymyalgia rheumatica; *RA*: Rheumatoid arthritis; *GIDM*: Glucocorticoid-induced diabetes mellitus; *SLE*: Systemic lupus erythematosus; *T2DM*: Type 2 diabetes mellitus

^*^Higher waist circumference, hypertriglyceridemia and higher METS-IR

^**^Greater number of organ system involvement, higher SLEDAI and SLICC-DI score

^◊^PED ≥ 5 mg/day (for the last 1 to 6 months) is associated with a risk increase ranging from 20 to 48%

^∇^Risk increases following a dose-dependent pattern

^∇∇^No exact percentage available

Regarding the risk factors for GIDM presentation in individuals with SLE, significant associations have been found with many variables, including both classic metabolic and disease-specific factors (Table 1) [78–81]. Among the classic metabolic risk factors, higher waist circumference [Odds ratio (OR): 1.10, 95% CI: 1.02–1.18], hypertriglyceridemia (OR: 4.25, 95% CI: 1.71–10.33) [80] and higher Metabolic Score for Insulin Resistance (METS-IR) (OR: 1.17, 95% CI 1.04–1.32) [78] have been identified to increase the occurrence of GIDM. In addition to metabolic components, SLE-specific factors are also linked to GIDM development. These include a greater number of organ system involvement (OR: 2.2, 95% CI: 1.06–4.58) [80], as well as a higher disease activity (SLEDAI score—OR: 1.25, 95% CI: 1.04–1.50) and organ damage (SLICC-DI score—OR: 4.93, 95% CI: 2.14–11.3) [78]. Moreover, a prednisone dose exceeding 1 mg/kg/day (OR: 7.6, 95% CI: 1.14–50.24) [80] and a concurrent use of mycophenolate mofetil (MMF) (Ha et al.- OR: 4.80, 95% CI: 1.32–17.45/ Zabihiyeganeh et al.- OR: 5.1, 95% CI: 1.1–23.25) have been shown as independent predictors of elevated GIDM risk [79, 81]. In contrast, HCQ was associated with a lower prevalence of GIDM, as found in two different studies demonstrating a statistically significant protective correlation between the antimalarial and GIDM (OR: 0.14, 95% CI: 0.02–0.85/ OR: 0.11, 95% CI: 0.01–0.75) [78, 80].

In conclusion, GIDM in SLE individuals ranges from 10–26%, with classic metabolic risk factors, disease-specific risk factors and disease modifying antirheumatic drugs (DMARDs) further affecting the risk (Table 1).

#### Rheumatoid arthritis

Evidence on the prevalence of GIDM in RA cohorts is unclear, with most studies reporting no significant association in patients free of classic metabolic risk factors (Table 1) [82–85]. In a sub-analysis of the BeSt study, 240 non-diabetic early RA individuals were evaluated to assess the effect of prednisone (median dose 7.5 mg/day) on glucose homeostasis. After adjustment for disease activity and effect over time, age and BMI, neither current (hyperglycemia-OR: 1.01, 98.3% CI: 0.98–1.04/ DM- HR: 1.07, 98.3% CI: 0.98–1.16) nor any previous (hyperglycemia-OR: 1.22, 98.3% CI: 0.90–1.64/ DM- HR: 0.72, 98.3% CI: 0.28–1.84) prednisone use was significantly associated with development of hyperglycemia or DM, whereas this was also the case for other prednisone variables, such as previous time on prednisone, cumulative prednisone dose, and maximum previous prednisone dose [84]. In another study, enrolling 140 RA patients with a minimum disease duration of 2 years and without prior DM diagnosis, the effect of GC on glucose tolerance, on insulin sensitivity and on β-cell function was evaluated [85]. They were divided in two groups based on GC use, the first (n = 58) treated with low-to-medium dose of GCs and the other (n = 88) including GC-naïve RA individuals [85]. There were no significant differences in glucose tolerance state, metabolic responses during OGTT (glucose, insulin and c-peptide levels) and insulin sensitivity [as assessed by HOMA-IR (homoeostatic model assessment for insulin resistance), MCRest (estimated metabolic clearance rate) and OGIS (oral glucose insulin sensitivity index) between the two groups, whereas for β-cell function, except for HOMA-β (homoeostatic model assessment of β-cell – Fasting index) being higher in GC users, all dynamic parameters were comparable [Insulinogenic Index (IGI), Disposition Index (DI), and AUC c-pep /AUC gluc ratio (area under the curve of c-peptide to AUC of glucose ratio)] [85]. Although cumulative or daily GC dose was associated with incident T2DM in univariate analyses [cumulative dose (g) – OR: 1.04, p = 0.002; daily dose (mg) – OR: 1.13, p = 0.048], this was less profound after adjustment for disease activity and patient characteristics analyses [cumulative dose (g) – OR: 1.02, p = 0.08; daily dose (mg) – OR: 1.11, p = 0.3] [85]. Along the same lines, an observational study assessing glucose metabolism in premenopausal females with RA, free of conventional metabolic risk factors, reported no difference between GC users vs non-users in insulin sensitivity indices, including Matsuda (ISIMAT), Cederholm (ISICED) and HOMA2%S (HOMA2 model estimate of insulin sensitivity) [82]. Additionally, in an RCT, 41 untreated early active RA patients were enrolled [83] receiving either 30 mg/day or 60 mg/day of prednisolone treatment [83]. Impaired glucose metabolism was defined as a fasting plasma glucose level of 5.6–6.9 mmol/l or a 2-h OGTT value of 7.8–11.0 mmol/l. Results indicated that, despite incidence of T2DM rose from 7 to 24%, short-term prednisolone therapy did not significantly affect glucose metabolism neither between the two different dose groups nor compared to their baseline status [83].

On the other hand, there is accumulating data from registry-based studies supporting the increased risk of GIDM in RA [55, 86]. In a cohort study investigating the risk of incident DM associated with the dosage and duration of oral GC therapy, 34,619 individuals with RA were enrolled [86]. Data regarding the two variables were extracted from the United Kingdom Clinical Practice Research Datalink (UK CPRD) and United States National Data Bank for Rheumatic Diseases (US NDB). In the former, DM diagnosis was based on read codes, at least 2 prescriptions for oral antidiabetic medication, or abnormal glucose blood tests, whereas in the latter, only patient self-reports were used [86]. GC therapy in individuals with RA was associated with a higher occurrence of DM compared to GC non-users in both registries (UK CPRD – HR: 1.30, 95% CI: 1.17–1.45/ US NDB– HR: 1.61, 95% CI: 1.37–1.89), with the risk significantly rising for doses exceeding 10 mg/day PED (UK CPRD – HR: 1.97, 95% CI: 1.61–2.40/ US NDB – HR: 2.24, 95% CI: 1.72–2.93) [86]. In addition, compared to non-use, taking 5 mg PED for the last 1, 3 and 6 months was significantly associated with increases on DM risk ranging from 20 to 48% (1 month – aHR: 1.20, 95% CI:1.11–1.29/ 6 months – aHR: 1.48, 95% CI: 1.33–1.64) [86]. Comparably, a population-based longitudinal analysis of electronic health records from the UK CPRD, including 28,365 individuals with RA, reported a dose-dependent risk of GIDM [55]. Specifically, current use of 5 mg/day increased the probability of DM by 1.66 times (aHR: 1.66, 95% CI: 1.37–2.02), while daily doses exceeding 25 mg indicated an aHR of 4.00 (aHR: 4.00, 95% CI: 3.08–5.21) when compared to non-users [55]. In conclusion, among RA individuals, while short-term administration of low to moderate GC doses (≤ 7.5–10 mg/day PED) appears to have minimal impact on glucose metabolism in those without classic metabolic risk factors, large registry-based studies indicate that higher cumulative or daily doses (> 10 mg/day) and duration significantly increase the risk of GIDM (Table 1).

#### Other ARDs

There is limited data addressing its incidence in individuals with PMR or GCA [55, 87]. In a meta-analysis of 25 studies from 1979 to 2017, the incidence proportion of GIDM in PMR and GCA was assessed [87]. The analysis indicated that the cumulative incidence of GIDM in individuals with PMR was 6% (95% CI: 0.03–0.09) and with GCA was 13% (95% CI: 0.09–0.17) (Table 1) [87]. Regarding the effect of GC dose on GIDM, a different study enrolling 32,593 patients diagnosed with PMR or GCA indicated that daily doses up to 5 mg doubled the risk of GIDM compared to non-users (aHR: 2.00, 95% CI: 1.79–2.23), while for each additional 10 mg/day the hazard further amplified (daily dose 5.0–14.9 mg- aHR: 2.29, 95% CI: 2.07–2.53/ daily dose 15.0–24.9- aHR: 3.14, 95% CI: 2.64–3.74) [55]. For doses ≥ 25 mg/day the risk of GIDM was approximately 4 times higher compared to non-users (aHR: 3.88, 95% CI: 3.20–4.71). Finally, the same study evaluated 6,082 patients diagnosed with various types of vasculitis, based on diagnostic codes, undergoing GC treatment [55]. The results showed an increased GIDM risk in GC-users compared to non-users, (aHR: 2.20, 95% CI: 1.79–2.71) in a dose-related pattern (daily dose 5.0–14.9 mg- aHR: 2.14, 95% CI: 1.57–2.90/ daily dose 15.0–24.9 mg—aHR: 3.04, 95% CI: 1.89–4.87/ daily dose ≥ 25 mg- aHR: 3.66, 95% CI: 2.21–6.06) [55].

Notably, there is currently no available data on GIDM or GIH occurrence and/or risk factors in individuals with other ARDs, such as Systemic sclerosis (SSc), pSS, idiopathic inflammatory myopathies or SpA, as no studies have been conducted in these populations to date. The occurrence and risk factors of GIDM across ARDs are summarized in Table 1, while the overall characteristics of the included studies are detailed in Supplementary Table 1.

## Discussion

Despite consistent evidence linking GC exposure to impaired glucose homeostasis, the true incidence of GIDM in ARD populations remains uncertain, with existing literature suggesting that GIDM is a real but underrecognized complication. In SLE, GIDM ranges from 10–26%, while in RA, the risk for GIDM appears to be dependent on the dose/duration of GC treatment and on the presence of classic metabolic risk factors. For other ARDs, including SSc, inflammatory myopathies, and SpA, such data are lacking, underscoring the need for further research on GC therapy. Future studies should focus on the dose-dependent adverse effects of GC treatment, ideally identifying a cut-off dose associated with GIDM, which could help establish standards of practice for close monitoring of at-risk individuals. This would also lay the groundwork for reconsidering what constitutes low- versus high-dose therapy, with implementable changes in nomenclature that could influence daily clinical practice and the perception of GC therapy. Different routes of administration should also be evaluated through both head-to-head clinical trials (e.g., oral versus IM GC therapy across different ARDs) and observational or registry-based studies. In the case of IM or IA GC injections, future research should explore different release patterns (rapid, intermediate or extended release) and their pharmacodynamic profiles in relation to GIDM risk. In addition, basic and translational research should further investigate the underlying mechanisms driving GIDM development in individuals with ARDs, contributing to the phenotyping of patients based on distinct tissue- and organ-specific pathways. In this context, linking distinct phenotypes (or endotypes) of the underlying disease (e.g. Individuals with PsA and concomitant metabolic syndrome or obesity) to the risk of GIDM may facilitate earlier identification and more effective preventive strategies. International rheumatology organizations (EULAR, ACR, APLAR) should prioritize initiatives in this area by setting research priorities (e.g., GC-focused study groups), fostering and funding collaborations (e.g., large multicenter, multidisciplinary studies), standardizing definitions, promoting physician education and awareness, and facilitating policy and advocacy.

To date, no specific recommendations from rheumatology organizations exist regarding the management of GIDM in individuals with ARDs following GC therapy. Management should be guided by a multidisciplinary approach, involving close collaboration between rheumatologists and endocrinologists. This allows for a personalized therapeutic strategy tailored to GC type, dose, duration, and regimen, as well as patient-specific factors such as age, renal and hepatic function, comorbidities, and pre-existing glucose tolerance. The choice between insulin therapy (first line) and classic oral hypoglycemic agents (including metformin and sulfonylureas) should be guided by the severity of hyperglycemia, underlying health status, and anticipated duration of GC therapy. The potential role of newer agents, including GLP-1 receptor agonists, should be considered in specific phenotypes (e.g., individuals with comorbid metabolic syndrome) in consultation with an expert due to their insulin-sensitizing and weight-reducing effects [88]. A pre-established monitoring plan, including frequent glucose assessments, should be implemented and adjusted according to inpatient or outpatient status. OGTT and HbA1c measurements are not routinely recommended for diagnosing GIDM in the acute setting, as they may be inaccurate or misleading [5]. In hospitalized patients with preexisting diabetes, blood glucose should be measured four times daily, whereas in non-diabetic patients, once-daily monitoring is sufficient after starting GC therapy [5]. For outpatients, a practical approach is to monitor blood glucose at home twice weekly in high-risk individuals [5]. Following the reduction or discontinuation of GC therapy, a corresponding tapering of antidiabetic treatment is typically warranted. Given the relatively rapid reversal of GIDM, antidiabetic therapy should generally be reduced within 24 h of GC cessation or dose reduction to minimize the risk of hypoglycemia. Despite this expected decline in glucose levels, continued blood glucose monitoring is essential to assess the patient’s glycemic trajectory and ensure safe transition back to baseline glucose control. In all cases, management should align with national recommendations.

Finally, since the rheumatologist is often the primary healthcare provider consulted by individuals with ARDs, providing guidance for GIDM prevention should be a core responsibility. Rheumatologists should inform patients about the risk of GIDM following initiation of GC therapy and provide counseling on dietary modifications (often in collaboration with a registered dietitian), weight management, and physical activity to help maintain glycemic control, as well as provide education about hyperglycemic symptoms which should prompt further testing. In patients with preexisting DM, physicians should ensure that a structured education program is in place, covering self-monitoring of blood glucose, insulin injection technique, hypoglycemia prevention, and general diabetes self-management.

To our knowledge, this is the first comprehensive synthesis of evidence on the epidemiology of GIDM in individuals with ARDs. The review provides a focused discussion of the underlying pathophysiology, glucocorticoid nomenclature and preparations, and an expanded examination of prevention and management strategies in this population. By highlighting key gaps in the existing literature, outlining research implications, and proposing future directions, we aim to raise awareness within the rheumatology community.

In conclusion, GIDM represents an important yet often overlooked complication of GC therapy, and rheumatologists should remain vigilant. Early identification of individuals at high risk is essential, as it enables timely preventive interventions and optimizes management outcomes.

## Supplementary Information

Below is the link to the electronic supplementary material.

Supplementary Material 1

## Data Availability

Not applicable.
