# Supplementary material for: Glucocorticoid-induced diabetes mellitus: mechanisms, risk factors, and clinical pathways with insights from autoimmune rheumatic diseases
Source: Rheumatol Int. 2026 Mar 26;46(4):66. doi: 10.1007/s00296-026-06099-z (PMC13017998; doi:10.1007/s00296-026-06099-z)
Supplement: Supplementary file 1 — Supplementary Material 1 [file 296_2026_6099_MOESM1_ESM.docx]

| **First Author (Year)** | **Country** | **Study Design** | **Disease(s)** | **Sample Size (n)** | **Glucocorticoid Regimen** | **Comparator** | **Outcome(s)** |
| --- | --- | --- | --- | --- | --- | --- | --- |
| Wu J. (2020)^55^ | United Kingdom | Population-based Longitudinal Cohort Study | • GCA or PMR (n=32,593)  • IBD (n=29,272)  • RA (n=28,365)  • Vasculitis (n=6,082)  • SLE (n=4,410) | 100,722 | **Systemic GCs:**  Oral, Intramuscular or Intra-articular: 17.1%  Inhaled or nasal: 10.7%  Rectal: 6.4%  Topical: 2.6%  **Daily prednisolone-equivalent dose categories:**  • Non-use  • 0-4.9 mg/day  • 5.0-4.9 mg/day  • 15.0-24.9 mg/day  • ≥25 mg/day | Period of non-GC use (exposed vs. unexposed person-time) | **GCA or PMR**  • GC dose >0–4.9 mg/day: HR=2.00, 95% CI [1.79-2.23]  • GC dose 5.0–14.9 mg/day: HR=2.29, 95% CI [2.07-2.53]  • GC dose 15.0–24.9 mg/day: HR=3.14, 95% CI [2.64-3.74]  • GC dose ≥25 mg/day: HR=3.88, 95% CI [3.20-4.71]  **IBD**  • GC dose >0–4.9 mg/day: HR=2.09, 95% CI [1.53-2.88]  • GC dose 5.0–14.9 mg/day: HR=2.34, 95% CI [1.93-2.84]  • GC dose 15.0–24.9 mg/day: HR=4.20, 95% CI [2.90-6.07]  • GC dose ≥25 mg/day: HR=5.05, 95% CI [4.09-6.24]  **RA**  • GC dose >0–4.9 mg/day: HR=1.66, 95% CI [1.37-2.02]  • GC dose 5.0–14.9 mg/day: HR=1.90, 95% CI [1.71-2.12]  • GC dose 15.0–24.9 mg/day: HR=3.07, 95% CI [2.28-4.14]  • GC dose ≥25 mg/day: HR=4.00, 95% CI [3.08-5.21]  **Vasculitis**  • GC dose 5.0–14.9 mg/day: HR=2.14, 95% CI [1.57-2.90]  • GC dose 15.0–24.9 mg/day: HR=3.04, 95% CI [1.89-4.87]  • GC dose ≥25 mg/day: HR=3.66, 95% CI [2.21-6.06]  **SLE**  • GC dose 5.0–14.9 mg/day: HR=2.67, 95% CI [1.94-3.68]  • GC dose 15.0–24.9 mg/day: HR=3.00, 95% CI [1.05-8.59]  • GC dose ≥25 mg/day: HR=6.63, 95% CI [3.55-12.36] |
| Dimas-Ramírez CA (2025)^78^ | Mexico | Observational Nested Case-control Study | SLE with biopsy-proven LN | 358;  **Case-Control analysis**: 105  • Cases: 35 (patients who developed GIDM or prediabetes)  • Controls: 70 (patients who did not develop GIDM) | Induction treatment with high-dose corticosteroids for LN  Steroid pulses: IV methylprednisolone 500-1000 mg | Control group: LN patients receiving GC induction but did not develop GIDM or prediabetes | **1. SLEDAI:** OR=1.25, 95% CI [1.04-1.50], p=0.01  **2. SLICC-DI:** OR=4.93, 95% CI [2.14-11.3], p<0.001  **3. METS-IR:** OR=1.17, 95% CI [1.04-1.32], p=0.009  **4. ΔMETS-IR at 6 months:** OR=1.20, 95% CI [1.03-1.39], p=0.01  **5. Antimalarial use:** OR=0.14, 95% CI [0.02-0.85], p=0.03 |
| Ha YJ (2011)^79^ | South Korea | Observational Retrospective Cohort Study | SLE | 127 | High-dose GC therapy defined as:  • Prednisolone 1 mg/kg/day for ≥4 weeks  • With or without one cycle of pulse therapy, consisted of three consecutive days of 1000 mg of intravenous methylprednisolone | SLE patients receiving high-dose GCs who did not develop GIDM | **1. GIDM incidence:** 12.6%  **2. Independent associated factors:**  • Older age (OR=1.08, 95% CI [1.03–1.13], p=0.003)  • Family history of diabetes (OR=10.29, 95% CI [2.33–45.54], p=0.002)  • Concurrent use of mycophenolate mofetil (OR=4.80, 95% CI [1.32–17.45], p=0.017) |
| Shahahir SS (2015)^80^ | Malaysia | Observational Cross-sectional Study | SLE | 100 | GC therapy | SLE patients receiving GCs who did not develop GIDM | **1. GIDM incidence:** 13%  **2. Independent associated factors**  • Prednisolone ≥ 1 mg/kg/day: β= 2.02; OR=7.6, 95% CI [1.14–50.24], p=0.04  • Number of system involvement: β= 0.80; OR=2.2, 95% CI [1.06–4.58], p=0.03  • Waist circumference: β= 0.10; OR=1.10, 95% CI [1.02–1.18], p=0.01  • Hypertriglyceridemia: β= 1.43; OR=4.25, 95% CI [1.71–10.33], p=0.002  **3. Hydroxychloroquine:** β= -2.28; OR=0.11, 95% CI [0.01–0.75], p=0.03 |
| Zabihi Yeganeh M. (2013)^81^ | Iran | Observational Cross-sectional Study | SLE | 81 | High-dose GC therapy defined as:  • Prednisolone equivalent ≥1 mg/kg/day  • With or without intravenous 1000 mg methylprednisolone cycle pulse therapy, consisted of three consecutive days | SLE patients receiving high-dose GCs who did not develop GIDM | **1. GIDM incidence:** 25.9%  **2. Independent associated factors:**  • Older age (OR=1.07, 95% CI [1.05–1.15], p=0.002)  • Family history of diabetes (OR=22.72, 95% CI [3.9–125], p>0.001)  • Concurrent use of mycophenolate mofetil (OR=5.1, 95% CI [1.1–23.25], p=0.035) |
| Penesová A. (2013)^82^ | Slovakia | Observational Cross-sectional Controlled Study | RA | 37;  • RA: 22  • Healthy controls: 15  *all participants were free of metabolic risk factors | Low-dose chronic GC therapy defined as:  • <8.5 mg/day of prednisone or equivalent  • Duration ≥ 2 years | • RA patients without GC therapy  • Healthy controls | There were no significant differences (p>0.05) in any insulin sensitivity indices between the 3 groups. |
| den Uyl D. (2012)^83^ | Netherlands | Randomized Controlled Single-blind Clinical Trial | Early active RA | 41;  • Prednisolone 30 mg/day: 20  • Prednisolone 60 mg/day: 21 | • Prednisolone 60 mg/day  OR  • Prednisolone 30 mg/day  • Duration: 7 days | • Prednisolone 60 mg/day group vs prednisolone 30 mg/day group  • Both groups were also compared to their baseline status | There were no significant differences (p>0.05) in glucose metabolism neither between the 2 groups nor compared to their baseline status. |
| van der Pol JA (2024)^84^ | Netherlands | Post-hoc Longitudinal Cohort Subanalysis of a Randomized Controlled Trial | Early RA | 504;  Ever prednisone: 240  Never prednisone: 264 | Group 3 - Tapered high dose of prednisone (concomitant methotrexate, sulfasalazine):  • Starting with 60mg/day  • Tapered to 7.5mg/day in 7 weeks  • Maintained for ≥28 weeks  • Gradually tapered further in case of sufficient response  The other 3 groups could add prednisone (by protocol), if required, with maximum dose at 7.5mg/day. | • No prednisone exposure  • Different cumulative doses  • Disease activity (DAS) levels | No association between prednisone use and:  • Glucose levels over time  • Hyperglycemia  • Development of diabetes |
| Hoes JN (2011)^85^ | Netherlands | Observational Cross-sectional Controlled Study | RA | • RA: 140  RA-GC (GC-naive): 82  RA+GC (GC users ≥3 months): 58  • Healthy controls: 50 | RA+GC: low-to-medium dose of GCs for ≥3 months | • RA-GC  • Healthy controls | **1. RA+GC and RA-GC had no significant differences in:**  • β-cell function  • Insulin sensitivity  **2. Cumulative or daily GC dose was not associated with incident T2DM in multivariate analyses:** • Cumulative dose (g): OR= 1.02, p=0.08 • Daily dose (mg): OR= 1.11, p=0.3 |
| Movahedi M. (2016)^86^ | United Kingdom and United States | Observational Retrospective Cohort Study | RA | 34,619;  • UK CPRD: 21,962  • US NDB: 12,657 | GC therapy | • RA patients without GC therapy | **1. GC therapy in individuals with RA was associated with a higher occurrence of DM compared to GC non-users in both registries:**  UK CPRD: HR=1.30, 95% CI [1.17-1.45]  US NDB: HR= 1.61, 95% CI [1.37-1.89]  **2. The risk further increased for doses exceeding 10 mg/day PED:**  UK CPRD: HR= 1.97, 95% CI [1.61-2.40]  US NDB: HR= 2.24, 95% CI [1.72-2.93]  **3. Compared to non-use, taking 5 mg PED for the last 1, 3 and 6 months was significantly associated with increases on DM risk ranging from 20% to 48%:**  1 month: aHR= 1.20, 95% CI [1.11-1.29]  3 months: aHR= 1.43, 95% CI [1.29-1.57]  6 months: aHR= 1.48, 95% CI [1.33-1.64] |
| Lai LYH (2017)^87^ | United Kingdom | Systematic Review and Meta-analysis | • PMR  • GCA | 3,743;  • PMR: 920  • GCA: 2,823 | GC therapy | N/A | **GIDM incidence:**  PMR: 6% (95% CI: 0.03-0.09, p=0.002)  GCA: 13% (95% CI: 0.09-0.17, p<0.0001) |

**Supplementary Table-1:** Characteristics of Studies Included Assessing GIDM in ARDs.

ARDs: Autoimmune Rheumatic Diseases; DAS: Disease activity score; GC: Glucocorticoid; GCA: Giant cell arteritis; GIDM: Glucocorticoid-Induced Diabetes Mellitus; IBD: Inflammatory bowel disease; LN: Lupus nephritis; METS-IR: Metabolic Score for Insulin Resistance; PED: Prednisolone equivalent dose; PMR: Polymyalgia rheumatica; RA: Rheumatoid arthritis; SLE: Systemic lupus erythematosus; SLEDAI: Systemic Lupus Erythematosus Disease Activity Index; SLICC-DI: Systemic Lupus International Collaborating Clinics Damage Index; UK CPRD: United Kingdom Clinical Practice Research Datalink; US NDB: United States National Data Bank for Rheumatic Diseases
